# Journal of Trauma Management & Outcomes: a new platform for interdisciplinary, outcome-oriented research in trauma

**DOI:** 10.1186/1752-2897-1-1

**Published:** 2007-11-26

**Authors:** Axel Ekkernkamp

**Affiliations:** 1 Dept of Trauma and Orthopaedic Surgery, Unfallkrankenhaus Berlin and University of Greifswald, Germany

Welcome to *Journal of Trauma Management & Outcomes *an exciting, new, online journal, specifically dedicated to the epidemiology, care, and outcomes of severely injured patients.

The journal willconsider articles on all aspects of trauma research, with a focus on interventions with proven efficacy or inefficacy in improving clinically relevant outcomes such as mortality, morbidity, quality of life, function, and costs. It will serve as a scientific platform for clinical researchers and practitioners involved in the different stages of caring for patients with musculoskeletal, visceral, and multiple injuries.

Whilst other established journals exist in this field, several factors convinced me, and the fine members of our international Editorial Board [1], to launch the *Journal of Trauma Management & Outcomes *together with BioMed Central.

First, the projections of the World Health Organization on changes in the rank order of the global burden of disease are simply alarming. If they prove true (and there is no reason to doubt the forecasts), trauma will rank third (!) among all causes for disability in 2020 worldwide [2] – needless to say that this, apart from the detrimental consequences for patients and their families, will drain the global health care budgets. Similar to other important health-care issues like cardiovascular diseases, cancer, and HIV, there is an urgent need to foster public awareness of the importance of trauma, and to establish effective prevention campaigns.

Mobility is an integral part of our life, but we also have to deal with its adverse effects. With the exponential growth of motorization in developing nations, especially in Asia, and the demand for more powerful and faster vehicles in the industrial countries, the face of road crashes is changing. Traffic accidents affect both the rich and the poor, but the consequences on health-related outcomes are very different. There are few socio-economic areas where inequities and heterogeneity in health care services have such a strong influence on mortality and morbidity, beginning with proper resuscitation on scene, and not ending with physical and mental rehabilitation.

Our interdisciplinary team had the opportunity of implementing accident research and prevention programs in Germany and Vietnam [3], and it is our responsibility to share our experience with other experts in the field.

This leads me to the second argument in favor of a new journal: Important biomedical information cannot change the world unless it is accessible to everybody. There are many highly reputed journals in the field, but few of them grant readers access to their full content. All articles published in *Journal of Trauma Management & Outcomes *are open access, meaning they are freely and universally accessible online. In addition, the authors hold copyright for their work and grant anyone the right to reproduce and disseminate the article, provided that it is correctly cited and no errors are introduced. The journal's articles are archived in PubMed Central, the US National Library of Medicine's full-text repository of life science literature, and also in other national repositories.

Publishing online only meets the requirements of a modern world, and the quick turnover of medical information. It also shortens the time from peer-review to publication. All articles will primarily be reviewed in-house, and, if suiting the scope of JTMO, sent out to two or more international peer-reviewers. Peer-review will be conducted in an open manner, which, to my and the Editorial Board's members opinion, increases transparency.

Apart from all these advantages, there is a little downer: article processing charges need to be covered by authors. This has, however, not impeded the stable growth in the number of articles available open-access, and taking part in this success story may clearly outweigh the inconvenience of paying for publishing. Also, there are many opportunities to apply for a waiver of processing charges that can be found on our website.

Finally, the findings from basic trauma research contributed importantly to the knowledge of human physiology and pathophysiology. However, the research efforts and discoveries on the cellular, genomic, and molecular level are not paralleled by a similar progress in clinical and population-based research on trauma. Many established diagnostic and therapeutic interventions in trauma care have not yet been proven by studies of high methodological rigor, and we currently know much more about the interplay of cytokines than the quality of life of severely injured patients. *JTMO *will not publish basic research, but target the key components of modern health care-function, quality of life, and costs.

I am thankful to the wonderful people at *BioMed Central *who helped us in getting the journal started, the members of the Editorial Board, and, of course, the authors of the first articles who made *JTMO *the journal of their choice.

I am pleased to lead this journal as the Editor-in-Chief, and I hope that the acronym *JTMO *will soon become widely known among authors and readers, for the sake and benefit of trauma patients in a world without borders.
